# High Abundances of Potentially Active Ammonia-Oxidizing Bacteria and Archaea in Oligotrophic, High-Altitude Lakes of the Sierra Nevada, California, USA

**DOI:** 10.1371/journal.pone.0111560

**Published:** 2014-11-17

**Authors:** Curtis J. Hayden, J. Michael Beman

**Affiliations:** Life and Environmental Sciences and Sierra Nevada Research Institute, University of California Merced, Merced, California, United States of America; Missouri University of Science and Technology, United States of America

## Abstract

Nitrification plays a central role in the nitrogen cycle by determining the oxidation state of nitrogen and its subsequent bioavailability and cycling. However, relatively little is known about the underlying ecology of the microbial communities that carry out nitrification in freshwater ecosystems—and particularly within high-altitude oligotrophic lakes, where nitrogen is frequently a limiting nutrient. We quantified ammonia-oxidizing archaea (AOA) and bacteria (AOB) in 9 high-altitude lakes (2289–3160 m) in the Sierra Nevada, California, USA, in relation to spatial and biogeochemical data. Based on their ammonia monooxygenase (*amoA*) genes, AOB and AOA were frequently detected. AOB were present in 88% of samples and were more abundant than AOA in all samples. Both groups showed >100 fold variation in abundance between different lakes, and were also variable through time within individual lakes. Nutrient concentrations (ammonium, nitrite, nitrate, and phosphate) were generally low but also varied across and within lakes, suggestive of active internal nutrient cycling; AOB abundance was significantly correlated with phosphate (r^2^ = 0.32, *p*<0.1), whereas AOA abundance was inversely correlated with lake elevation (r^2^ = 0.43, *p*<0.05). We also measured low rates of ammonia oxidation—indicating that AOB, AOA, or both, may be biogeochemically active in these oligotrophic ecosystems. Our data indicate that dynamic populations of AOB and AOA are found in oligotrophic, high-altitude, freshwater lakes.

## Introduction

Nitrogen (N) is an essential nutrient for all life, and its availability serves as a critical factor for the growth of individual organisms, community composition, and ecosystem primary productivity in freshwater lakes [1], [2]. In many ecosystems, N availability—both quantity and chemical form—is largely dictated by microbial communities, which transform inorganic N into bioavailable forms, and actively cycle N through oxidation-reduction (redox) processes. Phosphorus (P) typically limits primary production in freshwater [3], but both absolute amounts, and relative ratios, of N and P are highly variable due to variations in lake nutrient sources, as well as internal cycling by phytoplankton, zooplankton, and microbes [1], [4], [5]. In oligotrophic aquatic systems, in particular, differences in size, growth rate, and chemical form of available nutrients may favor microorganisms in competition with phytoplankton for N [6]. Microbial control of both N quantity and chemical form has important implications for the degree of eutrophication in these ecosystems, and the degree to which allocthonous N inputs (i.e. atmospheric pollutants) may affect oligotrophic lakes [7].

Within the microbial N cycle, nitrification is a two-step process that involves the aerobic oxidation of reduced inorganic N compounds (i.e. NH_3_/NH_4_^+^) to nitrite (NO_2_^-^) and the subsequent oxidation of NO_2_^-^ to nitrate (NO_3_^-^). Nitrification links the mineralization of N to its eventual removal as dinitrogen gas (N_2_) via either denitrification or anaerobic ammonium oxidation (anammox). The first step of nitrification is carried out by a few bacterial lineages within the *Beta-* and *Gamma-proteobacteria* and also by the archaeal phylum *Thaumarchaeota* (previously know as the group 1 *Crenarchaeota*) [8]. These ammonia-oxidizing bacteria (AOB) and ammonia-oxidizing archaea (AOA) use ammonia monoxygenase (AMO) to catalyze the oxidation of NH_4_^+^ to NO_2_^-^. As AOA were confirmed to be capable of ammonia oxidation only recently [9], the physical and chemical factors that control the abundance and function of these organisms, and their relative influence on nitrification rates, are not entirely understood—particularly in freshwater environments [10]–[13].

AOA, AOB, and nitrification have been examined within few freshwater lakes, yet AOA appear to be important and dynamic components of lake plankton and biogeochemical cycles: *Thaumarchaeota* are abundant [14], most appear to be AOA [15], and their populations fluctuate over time [15] and with depth [16]. AOB are found in lakes ranging from temperate eutrophic, to high-altitude oligotrophic, but in contrast to AOA, how AOB abundance varies in lakes through space and time is not well known [17]–[19]. The abundances of AOA and AOB can be controlled by differential sensitivities to temperature [20], pH [21], [22], ammonium concentrations [23], and light [24]—all of which may be relevant in high elevation lakes, but have not been examined. Nitrification varies with depth and time, and is quantitatively important within lake water columns [10], [25]–[27]—for example, Finlay et al. [25] showed that within-lake production of NO_3_^-^ through nitrification is the predominant source of NO_3_^-^ in Lake Superior. However, a lone study has measured both AOA and ammonia oxidation rates in freshwater lakes [10], and AOB have rarely been quantified in lakes [28]. We therefore know little about variations in AOA and AOB abundance and activity over time and across different lakes—let alone how AOA, AOB, and ammonia oxidation rates respond to changes in temperature, N availability, and other environmental factors within freshwater systems.

Of particular relevance are the potential inhibitory effects of light on nitrification: while these have been known for some time (reviewed by [29]), the relative effects of different wavelengths of light on AOA versus AOB, and in the field versus lab, are mixed. AOA appear highly sensitive to light in controlled experiments [24], [30]: the AOA *Nitrosopumilus maritimus* and *Nitrosotalea devanaterra* were inhibited by lower light levels than AOB, and showed little recovery of ammonia oxidation over 8/16 hour light/dark cycles [30]. French et al. [24] likewise found that ammonia oxidation by three freshwater AOA isolates was strongly inhibited by white and blue light, whereas an AOB isolate was inhibited only by blue light and recovered partial oxidation ability in the dark. Notably, all of the AOA isolates used in these studies have been recovered from sediments or soil, and it is possible that pelagic AOA are less light-sensitive—for example, Auguet and Casamayor [14] proposed that surface waters of mountain lakes are an archaeal ‘hotspot’ based on high crenarchaeal abundance in the neuston. AOA also actively express *amoA*, and nitrification is known to occur at least transiently, in the upper ocean [31]–[33].

**Figure 1:**
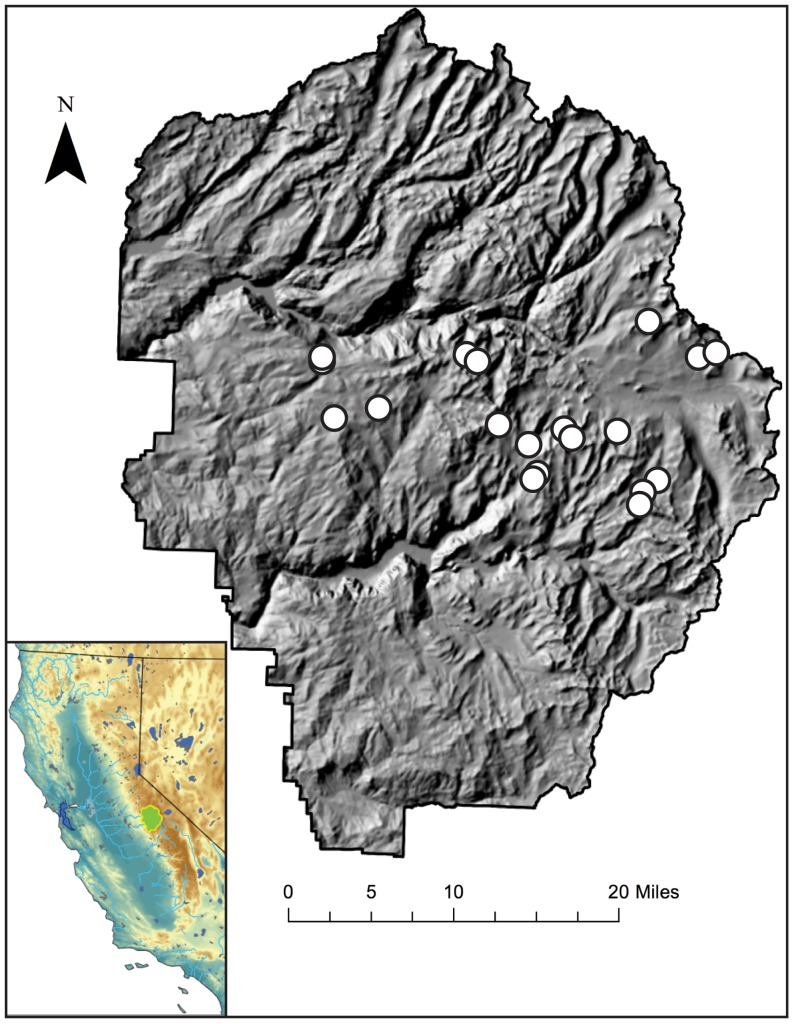
Sampling locations (white circles) in Yosemite National Park displayed on 10 m resolution elevation data from the United States Geological Survey National Elevation Dataset (http://nationalmap.gov/). Inset: location of Yosemite shown as the green shaded area within the state of California.

We quantified the abundance of AOA and AOB across a high-elevation lake transect in Yosemite National Park, in the Sierra Nevada mountain range, California, USA (Figure 1). In Sierra Nevada lakes, the differing susceptibility of AOA and AOB to photoinhibition could be a crucial factor in N cycling, as there is a strong natural increase in ultraviolet (UV) radiation with increasing elevation [26]. Moreover, these high-altitude lakes have relatively low light attenuation due to high transparency typical of oligotrophic aquatic ecosystems found at high elevations [26], [34]. Freshwater lakes are traditionally limited by phosphorous availability [35], but the availability of N is also a critical factor for primary productivity in aquatic ecosystems of the Sierra Nevada, where biological activity in ∼22% of lakes is strictly limited by N availability [36]. Internal N cycling may therefore play an important role in the overall productivity and structure of these freshwater ecosystems. We used natural variations in temperature, radiation, and N deposition, based on elevational and temporal variability between sampling sites, to examine the prevalence and abundance of AOA and AOB in high-altitude lakes.

## Materials and Methods

### Study Site

**Figure 2:**
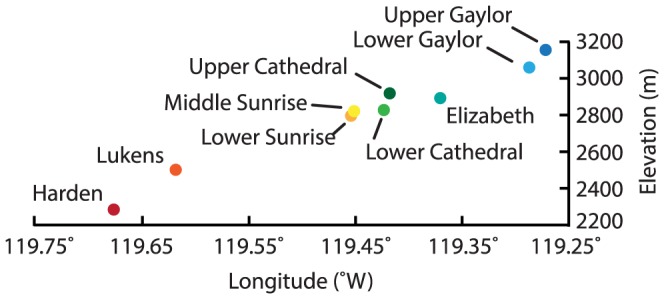
Lake elevation plotted against longitude for the nine lakes sampled in this study.

The Sierra Nevada (California) is a 400-mile long mountain range that gradually rises from the valley floor from west to east and reaches an apex of 3,000 to 4,200 meter peaks on its eastern edge (Figures 1 and 2). Vegetation along the mountain range is composed of grasslands and foothill woodlands at lower elevations, with a transition to mixed conifer forests, and then alpine meadows and lakes at higher elevations. Aquatic ecosystems in the Sierra Nevada are located downwind of urban and agricultural areas that emit high levels of N [37] and so experience elevated levels of N deposition [38]. This N deposition is known to increase N concentrations in lakes [7], and in the case of the Sierra Nevada, represents a large fraction of the N input to high-elevation lakes: Baron et al. [39] established a critical load threshold of 1.5 kg N ha^−1^ year^−1^ for high-elevation lakes located in Rocky Mountain National Park, yet current annual N loading in the Sierra Nevada (i.e. Emerald Lake Watershed, Sequoia National Park) ranges from 2.0 to 4.9 kg N ha^−1^ year^−1^ [40]. Moreover, the watersheds are high in granitic parent material and generally have thin soils; both characteristics cause aquatic ecosystems in the Sierra Nevada to have limited buffering capacity in terms of their ability to neutralize foreign chemical species [41]. Clow et al. [42] suggested that this property, coupled with high precipitation at high elevations, leads to high N loading at high elevations despite greater distances from emission sources. Our data are relevant to this as our selected sites range from 2300 m (Harden Lake) to 3160 m (Upper Gaylor Lake) (Table 1 and Figure 2) and our transect terminus is adjacent to the steep Sierra escarpment (Figure 1).

**Table 1:** Lake names, elevation, depth, and average nutrient concentrations.

Lake	Elevatio (m)	Dept (m)	Average PO_4_^3-^ (nM)	Average NH_4_^+^ (nM)	Average NO_2_^-^ (nM)	Average NO_3_^-^ (nM)
Harden	2289	4.5	80	15	44	297
Lukens	2506	6	98	32	41	374
Lower Sunrise	2801	5.5	74	13	38	432
Middle Sunrise	2826	5	116	25	52	212
Lower Cathedral	2832	10	73	13	28	281
Upper Cathedral	2923	3.5	91	20	67	419
Elizabeth	3050	9	84	10	36	232
Lower Gaylor	3064	11	81	41	33	398
Upper Gaylor	3160	7	79	20	26	307

### Sampling

Water samples were collected from lakes located in Yosemite National Park (YNP) during the 2012 summer. Nine lakes, which ranged in elevation from 2289 m to 3160 m and in depth from 4.5 m to 11 m (Table 1), were sampled in June, July, August, and September. Water samples were collected from the middle of each lake at 1 m depth using a Van Dorn water sampler (Lamotte); this depth was selected to include the effects of UV radiation and high photosynthetically active radiation (PAR). Duplicate water samples were filtered in the field onto 0.22 µm PVDF Membrane Filters (Millipore) using sterile 60 mL Polycarbonate syringes (Cole-Parmer). After filtration, filter membranes were packed into bead tubes (MP Biomedicals) containing 800 µL of Sucrose-Tris-EDTA. Filtrate was collected in 60 mL HDPE Bottles (Nalgene) for subsequent nutrient analysis. Samples were transported on ice to UC Merced's main campus or Yosemite Field Station (within hours of collection) and stored at −80° or −20°C until extraction of DNA and nutrient analysis. Samples were collected under USA National Park Service permit YOSE-2012-SCI-0111.

### DNA Extraction and quantification and real-time QPCR Analysis

DNA extraction followed Beman et al. [31] using Sucrose-Tris-EDTA (STE) lysis buffer, sodium dodecyl sulfate (SDS), and proteinase K with bead-beating. DNA was further purified using a DNeasy Blood & Tissue Extraction Kit (Qiagen) and resolubolized in 50 µL of ultra-pure DNA free water (Qiagen). DNA was quantified using a PicoGreen dsDNA quantification kit (Invitrogen) and an Mx3005P real-time thermocycler (Agilent Technologies). Total yield of DNA ranged from 23.5 to 922 ng.

Quantitative Polymerase Chain Reaction (QPCR) was used to quantify the abundance of *amoA* genes in lake samples. Primers, reaction chemistry, thermocycling, QPCR standards, quality control procedures, and data analysis exactly followed Beman et al. [31] and Beman et al. [43]. In brief, we used SYBR Green chemistry and the primers crenamoAF (5′-STAATGGTCTGGCTTAGACG-3′) and crenamoAR (5′-GCGGCCATCCATCTGTATGT-3′) for archaeal *amoA* (originally Arch-amoAF and R; [44]) and beta-amoA1F (5′-GGGGTTTCTACTGGTGGT-3′) and beta-amoA2R (5′-CCCCTCKGSAAAGCCTTCTTC-3′) for betaproteobacterial *amoA* (originally *amoA*-1F and *amoA*-2R [45]). QCPR efficiencies ranged from 89–91%, standard r^2^ values ranged from 0.98 to 0.99, and we tested for inhibition (which was not detected) by ‘spiking’ standards with samples [31], [43].

### Nutrient Analyses

Filtered lake water samples were analyzed for orthophosphate (µmol l^−1^) nitrite (µmol l^−1^) and nitrate (µmol l^−1^) using flow-injection analysis on a QuikChem 8000 (Zellweger Analytics, Inc.) at the University of California, Santa Barbara Marine Sciences Institute Analytical Laboratory (standard curve r^2^ = 0.999 for all assays). Filtered lake water samples were analyzed for ammonium (NH_4_^+^) following Holmes et al. [46]. 8 mL of filtered water was combined with 2 mL of reagent that consisted of 95% 0.1 M sodium tetraborate, 0.015 M O-pthaldialdehyde, 0.03 mM sodium sulfite, and 5% Ethanol. After aging, samples were measured in triplicate for fluorescence intensity using a fluorometer (Trilogy Laboratory Fluorometer, Turner Designs). Standards ranged from 31.2 to 186.8 nM and for different runs, standard curve r^2^ = 0.998–0.999.

### ^15^NH_4_^+^ oxidation rate measurements

Ammonia oxidation rates were measured by adding 99 atom percent (at%) ^15^NH_4_^+^ to a concentration of 200 nmol L^−1^, and measuring the accumulation of ^15^N label in the oxidized NO_2_^-^ + NO_3_^-^ pool after incubation for ∼24 hours [31], [47]. All samples were incubated within lakes to mimic in situ conditions as accurately as possible. *δ* ^15^N of NO_2_^-^ + NO_3_^-^ was measured at the UC Davis Stable Isotope Facility using the ‘denitrifier method’ [48], which produces N_2_O that can be analyzed on the mass spectrometer. Isotopic reference materials bracketed every 3–4 samples and coefficients of variation for these were 0.6%.

Initial at% enrichment of the substrate at the beginning of the experiment (*n*oNH_4_^+^, *see* Eq. 1) was calculated by isotope mass balance based on NH_4_ *^+^* concentrations determined fluorometrically [46] assuming that the ^15^N activity of unlabeled NH_4_^+^ was 0.3663 at% ^15^N. Rates of ammonia oxidation (*^15^R_ox_*) were calculated using equation 1 [31]:
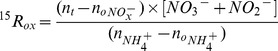
(eq.1) where *n_t_* is the at% ^15^N in the NO_3_^-^ + NO_2_^-^ pool measured at time *t*, *n*o_NOx_^-^, is the measured at% ^15^N of unlabeled NO_3_^-^ + NO_2_^-^, *n*o_NH4+_ is the initial at% enrichment of NH_4_^+^ at the beginning of the experiment, *n*_NH4+_ is at% ^15^N of NH_4_^+^ at time *t*, and [NO_3_^-^ + NO_2_^-^] is the concentration of the NO_x_^-^ pool.

### Statistical analyses

Statistical analyses were performed using the R statistical environment (http://www.r-project.org/) and the vegan package.

## Results and Discussion

**Figure 3:**
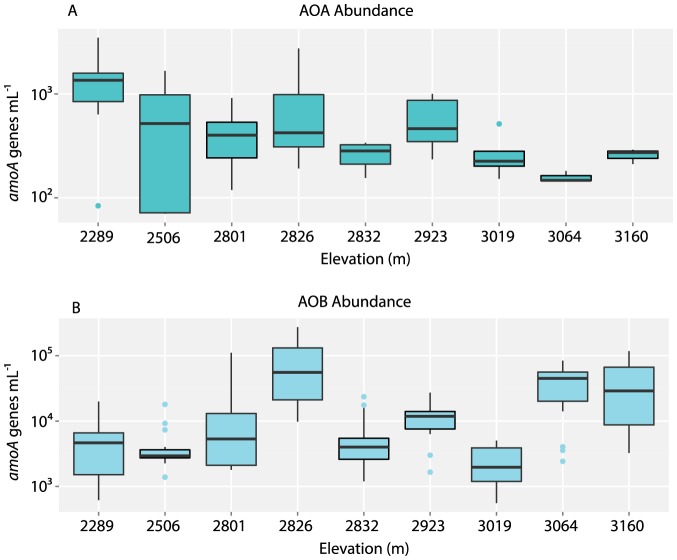
Boxplot comparison of number of *amoA* genes per milliliter (genes mL^−1^) for (A) ammonia-oxidizing archaea (AOA) and (B) ammonia-oxidizing bacteria (AOB). The vertical axes (logarithimic scale) denote the number of genes mL^−1^; the horizontal axes represent the elevation of the sampling sites, and are ordered from lowest elevation (Harden Lake) to highest elevation (Upper Gaylor Lake). In these plots, the box denotes the mean plus and minus one standard deviation; the line within the box represents the median value; and lines extending above and below the box span the full range of the data. Outliers were defined as sample values 1.5 times larger than the upper quartile and are represented by green and blue circles.

AOB *amoA* genes were detected in all lakes from June to September, and in 88% of samples, whereas AOA were found in 46% of all samples. AOB *amoA* gene copies ranged from 3.04×10^2^ to 2.07×10^5^ genes mL^−1^, however the majority (73%) of AOB values fell below 2×10^4^ genes mL^−1^, with values exceeding this occurring in June, August and September at high elevations (Figure 3B). AOA ranged from 0 to 4.58×10^3^ genes mL^−1^ (See Figure 3A) and there were zero instances where AOA outnumbered AOB. When AOA were detected, they were outnumbered by AOB by 2.8- to 1080-fold. Highest average abundances of both AOB and AOA were found in Middle Sunrise Lake (2826 m), while lowest average AOB abundances were found in Lukens Lake (2506 m), Lower Cathedral Lake (2832 m) and Lake Elizabeth (3019 m) (see Figure 3). For AOA, multiple lakes had low average values, including Lukens, Lower Cathedral, Elizabeth, and Lower Gaylor (3064 m). Over time, the highest numbers of *amoA* genes were present in September for AOB (4 out of 6 lakes above 2800 m), and in June for AOA (5 out of 6 lakes above 2800 m). AOB and AOA therefore appear to be present, and sometimes abundant, within the water columns of oligotrophic lakes in Yosemite National Park.

Previous studies that have quantified AOA abundance in high-altitude oligotrophic lakes reported *amoA* gene abundances as high as 3×10^4^ genes mL^−1^ [15], while AOB were below detection limits in the same lakes [49]. Our results indicate that AOB are more prevalent and abundant than AOA within oligotrophic lakes of the Sierra Nevada, CA, and AOB were dominant regardless of lake nutrient concentrations (see below), date of sampling, or lake elevation. This contrasts with earlier work that found AOA were more prevalent (detected via PCR but not quantified by QPCR) than AOB in oligotrophic lakes of the Tibetan plateau [18], and dominant in oligotrophic lakes of the Spanish Pyrenees [49]. In two contrasting lower elevation (231–825 m) lakes in France, Hugoni et al. [28] reported AOA dominance under low ammonium concentrations and oligotrophic conditions, whereas AOB were dominant in nutrient-rich waters [28]. The lakes sampled in Yosemite are uniformly nutrient-poor and have particularly low ammonium concentrations (all <75 nM), however our data indicate that AOB *amoA* genes are more abundant and prevalent than those from AOA. One explanation for AOB dominance in these lakes is that the *amoA* primers used to detect AOB and AOA may over- or under-estimate their abundances. However, the AOB primers used here are specific for betaproteobacterial AOB and are widely used [50]; the AOA primers amplify a wide range of AOA groups from water, sediments, and soils [44]. It is therefore unlikely that the AOB primers severely overestimate AOB *amoA* genes or that the AOA primers severely underestimate AOA *amoA* genes. Nor would this explain the prevalence of AOB, which were frequently detected. AOA were also detected in nearly half the samples—despite high light levels and oligotrophic conditions that are presumably hostile to nitrifiers in general.

Ultimately AOB and AOA must oxidize N to persist under oligotrophic conditions, and we suggest that their populations could be sustained by N fluxes that are not reflected in depleted nutrient pools: that is, NH_4_^+^ may be rapidly regenerated, assimilated, and/or oxidized, but because of high demand, does not accumulate in oligotrophic lakes. In fact, lakes are watershed ‘integrators’ [51] that can function as hotspots of N-cycling, including nitrification [14], [25], [42], [52]. In Yosemite, lake N loading increases with elevation, and NO_3_^-^ concentrations are correlated with modeled N deposition rates [42]. N deposition to Sierra watersheds occurs primarily as NH_4_^+^ [37] and this flux of N—which can be comparatively large in these oligotrophic ecosystems [53]—must have been nitrified at least once if it accumulates as NO_3_^-^. Ammonium concentrations therefore may not be the sole predictor for AOA and AOB abundances—elevation and nitrate concentrations may also be relevant—and we analyzed relationships between AOA and AOB and several types of spatial and nutrient concentration data using a variety of statistical approaches.

**Figure 4:**
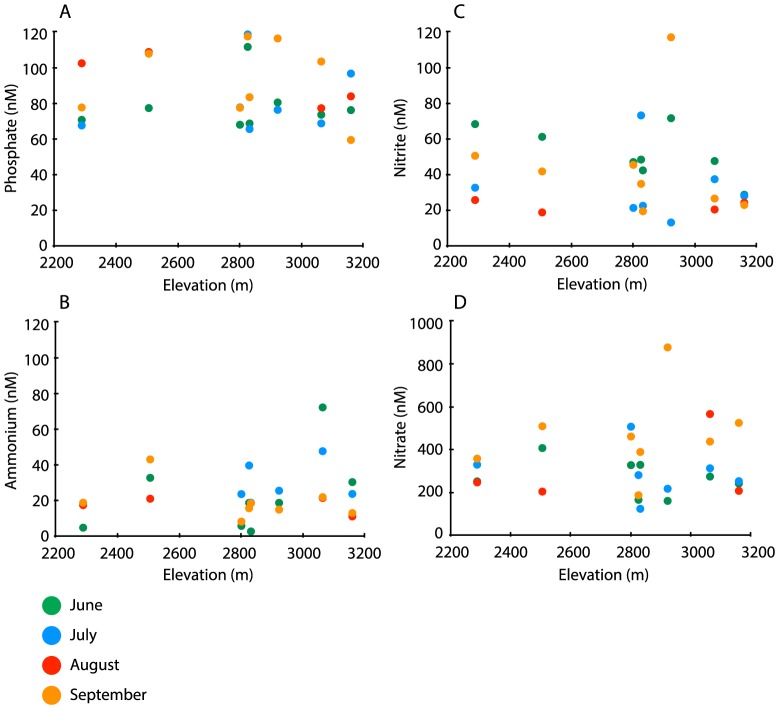
Variation in lake nutrient concentrations versus elevation for (A) phosphate (PO_4_^3-^), (B) ammonium (NH_4_^+^), (C) nitrite (NO_2_^-^), and (D) nitrate (NO_3_^-^). Vertical axes show nutrient concentrations in nanomolar (nM) plotted on a logarithmic scale, and colors denote month of sampling. Note that some samples have highly similar nutrient concentrations and fall nearly on top of one another.

During the sampling period NO_3_^-^ concentrations in all lakes ranged from 150 nM to 990 nM, PO_4_^3-^ concentrations from 60 nM to 120 nM, NO_2_^-^ concentrations from 20 nM to 120 nM, and NH_4_^+^ concentrations from 2.8 nM to 72 nM (Figure 4). As expected in aquatic ecosystems, NO_3_^-^ levels were higher than either PO_4_^3-^ or NO_2_^-^. We did not observe a significant trend with elevation (ANOVA *P*>0.05), but this could emerge with additional sampling. In nearly all lakes, the molar concentration of PO_4_^3-^ was higher than that of nitrite, which is typical, as NO_2_^-^ is quickly oxidized to NO_3_^-^ in the presence of oxygen [54]. Across our samples, AOB and AOA were significantly correlated with a few individual variables, including nutrient concentrations. For example, AOB abundance was most strongly correlated with PO_4_^3-^ concentrations (r^2^ = 0.32, *p*<0.1), consistent with work by Sundaweshar et al. [55] that showed P-limitation of N-cycling. AOB displayed an increasing trend with altitude, but this relationship was not significant (r^2^ = 0.21, *p* = 0.28); in contrast, AOA abundance was inversely correlated with altitude (r^2^ = 0.43, *p*<0.05). Both groups showed wide variation in Middle Sunrise Lake at 2826 m. These patterns are evident in Figure 3, where AOB were notably more abundant—but also variable—in the Gaylor Lakes at >3000 m elevation, whereas AOA were more abundant and variable at lower elevations. For all samples collected in the three lakes >3000 m elevation, AOA were only detected four times. This inverse relationship between AOA abundance and increasing elevation could reflect the effect of increased UV radiation at higher elevations, or other factors that vary with elevation.

**Figure 5:**
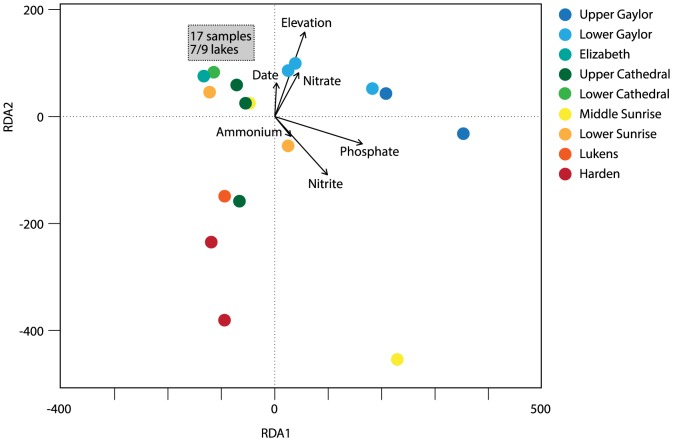
Bi-plot of redundancy analysis of bacterial community composition. Color key denotes different lakes, and arrows denote biplot scores for the constraining variables. The grey box encompasses 17 samples that fall within a narrow range of each other and are not visually distinguishable on the bi-plot; this includes 1–2 samples from every lake except Middle Sunrise and Upper Cathedral Lake. One sample from Middle Sunrise Lake with extremely high AOB *amoA* gene abundance is not shown, as it falls much farther along the RDA1 and RDA2 axes.

We therefore used redundancy analysis (RDA) to analyze multivariate relationships between AOA and AOB abundance and spatial (elevation and longitude), temporal (sampling date) and environmental (NO_3_^-^, NO_2_^-^, NH_4_^+^ and PO_4_^3-^) data (Figure 5). 33% of the variability in AOA and AOB abundance was explained by these data, with nutrient concentrations accounting for 24% of the variability in AOA and AOB abundance, and site location and sampling date accounting for 9% of the variability. Collectively, nutrients explain nearly a quarter of the variation in AOA and AOB abundance in these lakes, and this includes ammonium, as well as nitrate, nitrite, and phosphate, concentrations. The modest percentage of constrained variance overall indicates that AOA and AOB populations are affected by other, un-measured factors, or this may reflect stochastic variation in populations. In lakes, AOB and AOA abundance may be modified by active growth, but also by transport of cells into lakes via air, water, or suspended particles; competition with other organisms for ammonium; and trophic interactions, such as grazing, viral infection, and lysis. None of these have been directly investigated.

**Figure 6:**
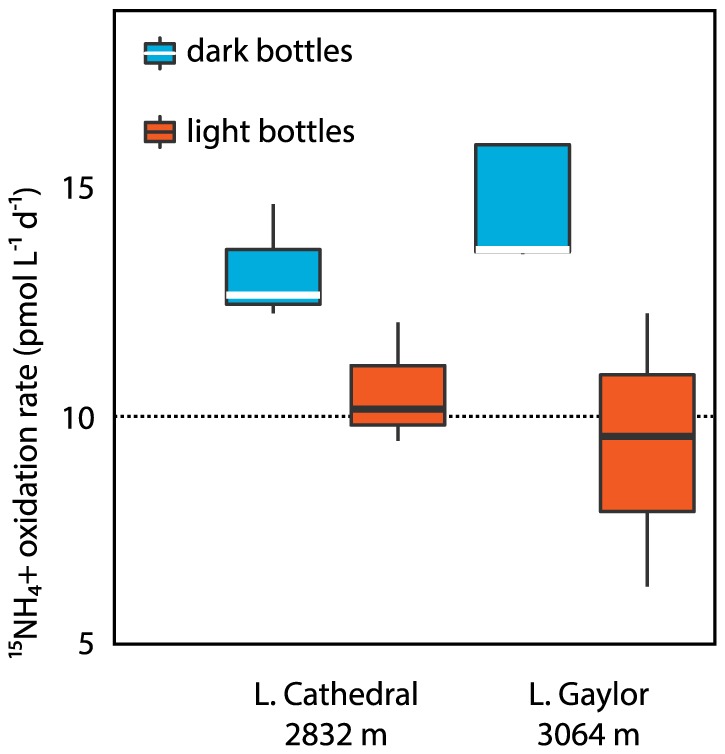
Boxplot comparison of calculated ^15^NH_4_^+^ oxidation rates measured in Lower Cathedral and Lower Gaylor lakes. The vertical axis shows ^15^NH ^+^ oxidation rates (pmol L^−1^ D^−1^). The blue bars represent dark bottle incubations that were incubated under zero-light conditions, while the orange bars represent samples that were exposed to ambient light over a 24-hour cycle during in-situ incubation at the sampling site. The dashed line notes the detection limit. In these plots, the box denotes the mean plus and minus one standard deviation; the line within the box represents the median value; and lines extending above and below the box span the full range of the data.

The prevalence and abundances of AOA and AOB suggest that active N cycling and nitrification may be occurring in high-altitude lakes, but is surprising given that high-altitude oligotrophic lakes have low N concentrations and experience high light levels. To determine whether AOA and AOB may be active under these conditions, we performed ^15^NH_4_^+^ incubations to detect ammonia oxidation rates in three lakes that span a range of elevations and AOB/AOA abundances (Lukens Lake, Lower Cathedral Lake and Lower Gaylor Lake). These were conducted in both light and dark bottles that were incubated within the lakes under *in situ* conditions. At low N concentrations found in oligotrophic waters, measuring ammonia oxidation is extremely challenging due to multiple factors: (1) measuring low-level N concentrations is difficult, and uncertainties in basic nutrient concentration values can strongly affect rate calculations; (2) addition of ^15^N label can significantly increase N concentrations and potentially introduce biases; (3) rates are expected to be low due to low N concentrations; and (4) measuring accurate isotopic values becomes difficult, which either introduces errors in the measurement or requires addition of N ‘carrier’—yet this can make detection of low rates difficult or impossible. For these reasons, we propagated uncertainties in nutrient measurements and isotopic values through our calculations, and found that rates were above detection (limit  = 10.2 pmol L^−1^ d^−1^) only in dark bottles in Lower Cathedral Lake and Lower Gaylor Lake—where they were extremely low (Figure 6). Ammonia oxidation was below the limits of detection in most light bottles in these lakes, as well as in Lukens Lake. That rates were undetectable under *in situ* light levels is consistent with light inhibition of ammonia oxidation, but more importantly indicates that AOB and AOA were inactive—or active at extremely low levels—at the time and location of sampling. Our data do suggest that they respond to changing light conditions and oxidize ammonia at low levels in completely darkened bottles. This expands the range of habitats in which ammonia oxidation can potentially occur to include surface waters of high-altitude, oligotrophic, freshwater lakes—but when and where these organisms are active at appreciable levels is not yet clear.

AOB and AOA were frequently present, varied in abundance between lakes, and fluctuated over time within individual lakes. The presence of AOA is consistent with the idea that they are adapted to the low-nutrient conditions that are characteristic of these lakes—for example, the marine AOA *Nitrosopumilus maritimus* has a remarkably high affinity for ammonia and appears to be adapted to life under extreme nutrient limitation [23]. However, AOB were more commonly detected and were more abundant than AOA under all sampled conditions; this fits with the recent idea that AOB are more light-tolerant than AOA [24], [30]. High altitude lakes could also experience higher fluxes of N that would periodically favor AOB [42]. Both AOA and AOB may contribute to nitrification in freshwater oligotrophic lakes—as we detected low rates of ammonia oxidation in darkened bottles—but it is clear that they were inactive, or active only at low levels, during our incubations. Our data therefore add to the limited information available on microbial contributions to N cycling in lakes—particularly for AOA/AOB and nitrification—but additional work should expand these approaches to additional lakes and additional sampling periods. Nitrification under ice could be important during winter, for example, and nitrification may be especially significant following spring snowmelt, when pulses of N likely enter these lakes and may be metabolized by microbes. Altogether our findings are indicative of dynamic microbial communities and internal N cycling in high-altitude lakes.
